# Investigation of Biopsychosocial Status, Fatigue, Sleep Quality, Alexithymia, Cognitive Functions, and Quality of Life in Behçet’s Disease

**DOI:** 10.31138/mjr.290823.ib

**Published:** 2023-08-29

**Authors:** Elif Gur Kabul, Sinem Yenil, Firdevs Ulutas, Bilge Basakci Calik, Veli Cobankara

**Affiliations:** 1 Faculty of Health Sciences, Physiotherapy and Rehabilitation, Usak University, Usak, Turkey,; 2 Faculty of Physiotherapy and Rehabilitation, Pamukkale University, Denizli, Turkey,; 3 Department of Rheumatology, Medical Faculty, Pamukkale University, Denizli, Turkey

**Keywords:** Behçet’s disease, anxiety, fatigue, cognition, sleep quality

## Abstract

**Objective:**

The aim of this study was to compare the effects of Behçet’s disease in terms of anxiety, biopsychosocial status, fatigue, sleep quality, alexithymia, cognitive level, and quality of life according to major and minor organ involvement.

**Methods:**

The study was planned as a single-centre cohort study. Fifty patients diagnosed with Behçet’s (mean age 43±11.96 years) were included in the study. The patients were divided into two groups as major organ involvement (uveitis, neuro-Behçet’s, or vascular type Behçet’s disease) and minor organ involvement (mucocutaneous type Behçet’s disease). Biopsychosocial status was evaluated with Biopsychosocial Questionnaire (BETY-BQ), anxiety with Beck Anxiety Inventory (BAI), fatigue with Multidimensional Assessment of Fatigue (MAF) Scale, sleep quality with Pittsburgh Sleep Quality Index (PSQI), alexithymia with Toronto Alexithymia Scale-20 (TAS-20), cognition level with Mini-Mental State Examination (MMSE), and quality of life with Short Form-36 (SF-36).

**Results:**

In the comparison according to minor and major organ involvement, there was no significant difference between the groups in BETY-BQ, BAI, MAF, PSQI, TAS-20, MMSE and SF-36 (p>0.05).

**Conclusion:**

Behçet’s disease negatively effects in parameters such as biopsychosocial status, fatigue, sleep quality, alexithymia and quality of life. The presence of major or minor organ involvement in the patients did not change these negative effects.

## INTRODUCTION

Behçet’s disease is a vasculitis that causes genital ulcers, eye and skin, joint, nervous system, cardiovascular, and gastrointestinal system lesions due to multisystemic inflammation. Although the aetiopathogenesis is not known exactly, there is a role of auto-immunity. The disease occurs in men with more severe forms.^1^ The disease can be divided into major and minor organ involvement according to organ involvement. Uveitis, neuro-Behçet’s and vascular type Behçet’s are called major organ involvement, while muco-cutaneous type is called minor organ involvement.^2^

Musculoskeletal symptoms such as arthritis, which can be observed in Behçet’s disease, cause chronic pain.^3^ It can also affect physical function and activity levels.^4^ In addition, functional deficiencies and stressful life due to the disease process affect the level of anxiety. Chronic stress suppresses the immune system.^5^ Thus, disease-related symptoms may increase in patients. One of the problems caused by chronic stress and pain could be poor sleep quality.^6^ At the same time, fatigue caused by chronic disease is another important problem in inflammatory diseases.^7^ Behçet’s disease can also affect the central nervous system and this disease may be accompanied by alexithymia.^8^ There may be neurocognitive changes in addition to alexithymia.^9^ Eye and skin involvement can affect daily life activities, social participation, and sexual life.^10^ Considering that it is a chronic immunoinflammatory disease with periods of exacerbation and remission, negative impact on quality of life is an expected result.^11^

The aim of this study was to compare the effects of Behçet’s disease in terms of anxiety, biopsychosocial status, fatigue, sleep quality, alexithymia, cognitive level, and quality of life according to major and minor organ involvement.

## MATERIALS AND METHODS

### Trial design

This study was designed as a single-centre cohort study. For the sample of the study, all Behçet’s patients followed by the Pamukkale University Rheumatology Clinic were reached, and all those who volunteered to participate and met the inclusion criteria were evaluated.

### Participants

Fifty patients with a mean age of 43±11.96 years who were followed by Pamukkale University Rheumatology Clinic and diagnosed with Behçet’s by the same rheumatologist were included in the study. The patients were divided into two groups as major organ involvement (uveitis, neuro-Behçet’s or vascular type Behçet’s disease) and minor organ involvement (mucocutaneous type Behçet’s disease).

Inclusion criteria: Diagnosed with Behçet’s according to the International Criteria for Behçet’s Disease, being in the age range of 20–65 years, volunteer to participate in the study. Exclusion criteria: concomitant autoimmune or inflammatory disease or any other additional neurological or orthopaedic disease that would affect assessments. The study was conducted in accordance with the ethical principles of the Declaration of Helsinki and ethical approval was obtained from Pamukkale University Non-Interventional Clinical Research Ethics Committee at the meeting dated 06.07.2022 and numbered 09. The patients included in the study were given detailed information about the study and their written informed consents was obtained.

### Evaluations

The Behçet’s patients were evaluated separately by the same rheumatologist and physiotherapist in approximately 40 minutes by face-to-face interview method on the day of appointment. The patients were asked to fill out the questionnaires themselves. However, the physiotherapist was with the patients while filling out the questionnaires and made necessary explanations for anything patients did not understand.

Demographic and disease-related data of the patients were recorded. Biopsychosocial status was evaluated with Biopsychosocial Questionnaire, anxiety with Beck Anxiety Inventory, fatigue with Multidimensional Assessment of Fatigue (MAF) Scale, cognition states with Mini-Mental State Examination, sleep quality with Pittsburgh Sleep Quality Index, alexithymia with Toronto Alexithymia Scale-20 and quality of life with Short Form-36.

#### Biopsychosocial Questionnaire (BETY-BQ)

The BETY-BQ evaluate the biopsychosocial status of rheumatic diseases patients with 30 items. Higher scores indicate a poor biopsychosocial status (Cronbach’s alpha value is 0.88).^12^

#### Beck Anxiety Inventory (BAI)

BAI consists of 21 items evaluating anxiety symptoms on a four-point Likert-type scale (0=“not at all” to 3=“severely”). The score of 0-21 indicates low; score of 22-35 indicates moderate, score of 36 and above indicates potentially concerning levels of anxiety (Cronbach’s alpha value is 0.92).^13^

Multidimensional Assessment of Fatigue (MAF) Scale MAF examines fatigue in four dimensions, including the degree and severity, distress, time and its effect on activities of daily living in the last 7 days. Scores range from 1 (no fatigue) to 50 (severe fatigue) (Internal Consistency Reliability was 0.90, Intraclass Correlation Coefficient reliability was 0.96).^14,15^

#### Mini-Mental State Examination (MMSE)

MMSE is an easy-to-apply questionnaire that is generally used to determine the level of cognitive impairment. It includes six categories: orientation; registration; attention and calculation; recall; language; and copying. The score of 24–30 indicates no cognitive impairment, the score of 18–23 indicates mild cognitive impairment, the score of 0–17 indicates severe cognitive impairment (Interrater reliability analysis showed high correlation was 0,99 and kappa value was 0,92).^16,17^

#### Pittsburgh Sleep Quality Index (PSQI)

PSQI consists of 19 questions and 7 components (subjective sleep quality, sleep latency, sleep duration, sleep efficiency, sleep disturbance, use of sleep medication, daytime dysfunction). Total score ranged from 0 to 21, with 0–4 indicating “good” sleep and 5–21 indicating “poor” sleep (kappa value was 0.75).^18^

#### Toronto Alexithymia Scale-20 (TAS-20)

It is a self-assessment scale consisting of 20 items. Each item is scored on a 5-point Likert-type scale (1=strongly disagree; 5=strongly agree). Scoring ranged from 20 to 100. The score of 0–51 indicates no alexithymia, the score of 52–60 indicates possible alexithymia, the score of 61–100 indicates alexithymia present (Cronbach alpha was 0.78).^19,20^

#### Short Form-36 (SF-36)

SF-36 had eight health concepts: physical functioning, bodily pain, role limitations due to physical health problems, role limitations due to personal or emotional problems, emotional well-being, social functioning, energy/fatigue, and general health perceptions. It consisted of 36 items. Each health concept was scored between 0 and 100 points, and high score indicates good health (validity coefficients was 0.79 for Physical component, and 1.02 for mental component).^21^

### Statistical analysis

Data were analysed using the SPSS (SPSS Inc., Chicago, IL, USA) program. For the sample of the study, all Behçet’s patients followed by the Pamukkale University Rheumatology Clinic were reached, and all those who volunteered to participate and met the inclusion criteria were included. Descriptive analyses were given as mean ± standard deviation, median, interquartile range, percent, minimum, and maximum.

For comparisons between groups, the Independent Samples T test was used if parametric conditions were met, and the Mann-Whitney U test was used if not. The significance level was accepted as p<0.05.

## RESULTS

### Recruitment

The time frame for patient recruitment and data collection was July 2022- November 2022.

### Baseline data

Demographic and disease-related data of the patients are given in **Table 1**. Descriptive data of the outcome measures are in **Table 2**. There was no significant difference between the groups separated according to major and minor organ involvement in terms of age, body mass index, disease duration and laboratory parameters (p>0.05). The pharmacological treatments received by Behçet’s patients were divided into 3 groups (first line oral therapy, biological therapy and no therapy), and there was a significant difference between major minor organ involvement (p:0.018) (**Table 3**).

**Table 1.** Demographic and disease-related data**.**

	**Behçet’s disease (n=50)**
	**Mean±SD**

Age (years)	43 *± 11.96*

BMI (kg/m^2^)	26.69 ± 4.56

Disease duration (years)	9.82 ± 8.05

Gender [n (%)]	
Female	23 (46)
Male	27 (54)

Disease type [n (%)]	
Mucocutaneous	23 (46)
Neuro-Behcet	9 (18)
Vascular	6 (12)
Uveitis	12 (24)

Exercise Habits [n (%)]	
Yes	20 (40)
No	30 (60

Beck Anxiety Inventory [n (% )]	
Low	36 (72)
Moderate	9 (18)
Potentially Concerning Levels	5 (10)

Biopsychosocial Questionnaire (BETY-BQ) [n (%)]	26 (52.0)
≥28	24 (48.0)
<28	

Multidimensional Assessment of Fatigue (MAF) Scale [n (%)]	
≥23	25 (50)
<23	25 (50)

Mini-Mental State Examination [n (%)]	43 (86)
No cognitive impairment	7 (14)
Mild cognitive impairment	0 (0)
Severe cognitive impairment	

Pittsburgh Sleep Quality Index [n (%)]	46 (92)
Poor Sleep	4 (8)
Good Sleep	

Toronto Alexithymia Scale-20 [n (%)]	
No alexithymia	10 (20)
Possible alexitymia	14 (28)
Alexitymia present	26 (52)

Short Form-36[n (%)]	
≥65	25 (50)
<65	25 (50)

**Table 2.** Descriptive data of the outcome measures.

	**Mean ± Standard deviation**
	**Minimum-Maximum**

Beck Anxiety	15.62 ± 13.05
Inventory	(0.00-51.00)

Biopsychosocial	37.60 ± 25.78
Questionnaire (BETY-BQ)	(0.00-100.00)

Multidimensional	22.64 ± 13.18
Assessment of Fatigue (MAF) Scale	(1.00-44.00)

Mini-Mental State	26.40 ± 2.53
Examination	(21.00-30.00)

Pittsburgh Sleep	8.18 ± 3.57
Quality Index	(1.00-16.00)

Toronto Alexithymia	60.86 ± 11.09
Scale-20	(33.00-86.00)

Short Form-36 Total	53.23 ± 23.06
	(11.44-92.88)

Short Form-36	55.57±24.73
Physical component	(7.50-96.25)

Short Form-36	56.89±24.14
Mental component	(5.00-92.00)

**Table 3.** Comparison of data between minor and major organ involvement in patients with Behçet’s disease.

	**Minor organ involvement (n:23)**	**Major organ involvement (n:27)**	**p**
	**n (%)**	**n (%)**	
Pharmacological treatment	16 (69.6)	11 (40.7)	0.018 †
-first line oral therapy			
**-** biological therapy	4 (17.4)	15 (55.6)	
**-**no therapy	3 (13.0)	1 (3.7)	
	**Mean ±SD**	**Mean ±SD**	
Laboratory parameters-AST	18.26±5.80	17.91±6.10	0.844 **
-ALT	19.91±10.17	22.85±14.44	0.755 *
-C-reactive protein	7.67±17.22	5.36±8.16	1.000 *
- Erythrocyte sedimentation rate	17.13±11.84	14.53±11.92	0.393 *
- Glucose	94.82±12.29	103.59±49.50	0.585 *
-Urea	27.94±6.85	28.42±8.47	0.744 *
-Creatinine	0.84±0.16	0.82±0.16	0.747 **
Age (years)	43.26 ± 11.92	42.77 ± 12.21	0.88 **
BMI (kg/m^2^)	26.80 ± 5.20	26.60 ± 4.04	0.87 **
Disease duration (years)	9.73 ± 9.16	9.88 ± 7.14	0.59 *
Beck Anxiety Inventory	13.43 ± 11.80	17.48 ± 13.97	0.37 *
Biopsychosocial Questionnaire (BETY-BQ)	36.04 ± 25.63	38.92 ± 26.31	0.67 *
Multidimensional Assessment of Fatigue (MAF) Scale	21.54 ± 12.60	23.59 ± 13.81	0.58 **
Mini-Mental State Examination	26.39 ± 2.57	26.40 ± 2.56	0.98 **
Pittsburgh Sleep Quality Index	8.30 ± 3.13	8.07 ± 3.97	0.69 *
Toronto Alexithymia Scale-20	60.30 ± 9.18	61.33 ± 12.64	0.74 **
Short Form-36 Total	59.82 ± 25.55	53.17 ± 2.70	0.24 *
Short Form-36 Physical component	60.00±25.77	51.80±23.64	0.235 *
Short Form-36 Mental component	59.65±27.54	54.53±21.07	0.289 *

AST: Aspartate Aminotransferase Test; ALT: Alanine Aminotransferase Test;

† Chi-Square Test;

* Mann-Whitney U Test;

** The Independent Samples T Test.

The median value of BETY-BQ is 28, the median value of MAF Scale is 23, and the median value of SF-36 is 65. 36% of patients had low anxiety, 9% had moderate anxiety and 5% had potentially concerning levels of anxiety. 52% of the patients had a score above the median value of BETY-BQ, that is poor biopsychosocial status. 50% of the patients had a score above the median value of the MAF Scale, that is high fatigue. The sleep quality of 92% of the patients was poor. Possible alexithymia was present in 28% of the patients, and alexithymia in 52%. 50% of patients were found to have a score below the median value of SF-36, that is low quality of life (**Table 1**).

### Comparison between the groups

In the comparison made according to minor and major organ involvement, there was no significant difference between the groups in BETY-BQ, BAI, MAF, PSQI, TAS-20, MMSE and SF-36 (p>0.05) (**Table 3**).

## DISCUSSION

The results of this study were that most of the Behçet’s patients had poor biopsychosocial status, high fatigue levels, alexithymia, and poor sleep and life quality. In addition, the results of patients with major or minor organ involvement were similar.

Studies have shown that anxiety and depression are common psychiatric disorders in Behçet’s patients and in some cases precede the onset of Behçet’s typical symptoms.^22^ Functional deficiencies and stressful life due to the disease process affect the level of anxiety and depression. The resulting chronic stress suppresses the immune system.^5^ This may lead to an increase in disease-related symptoms in patients. Studies have found that Behçet’s patients have an increase in anxiety, depression and fatigue scores compared to the healthy control group.^23,24^ Sandikci et al. reported that pain directly affects the psychosocial state. There is a positive relationship between headache and anxiety.^23^ These psychological problems may result from difficulties in living and coping with symptoms, sexual difficulties, sleep changes, stress, cognitive impairment, treatment changes, drug side effects, fatigue, pain, and inability to accept the diagnosis.^24^ In this study, we observed that 36% of Behçet’s patients had low anxiety, 9% had moderate anxiety, and 5% had potentially concerning levels of anxiety. Although the Behçet’s patients had major or minor organ involvement, there was no difference in anxiety. These results show that even if the physical symptoms decrease in these patients despite drug treatments, the psychological impact may continue. For this reason, we believe that supporting these patients with non-pharmacological treatment approaches together with pharmacological treatments can contribute to improving anxiety.

In a study conducted by Özgüler et al. based on patients’ self-reports, Behçet’s patients frequently mentioned the complaint of inability to walk due to joint pain and ulcers on the feet. The loss of mobility has affected patients’ daily activities, including work restrictions or a reduction in spending time with friends and family. In addition, patients reported that they felt upset or angry, especially with loss of mobility, fatigue, or side effects of treatments. The patients expressed fear or concern about how their symptoms would progress, that the symptoms would become more severe or that the patients could become dysfunctional.^10^ All these results show the biopsychosocial impact of the disease in Behçet’s patients. Therefore, in this study, we evaluated the biopsychosocial status of Behçet’s patients with the BETY scale, which was developed specifically with the opinions of patients with rheumatic disease.^12^ One of the results of this study is that patients are also affected biopsychosocially. We think that it will be important for the treatment team to adopt the biopsychosocial model in order to overcome these problems.

Fatigue was found to be more common in Behçet’s disease patients compared to healthy controls.^23,24^ Arthritis and increased inflammation, even anxiety and quality of life, increase fatigue.^23^ Ilhan et al. reported that mental symptoms such as depression and anxiety, and physical dysfunction were significantly associated with fatigue in Behçet’s patients.^7^ Behçet’s patients said that illness or being sick left them “tired”.^10^ This fatigue can cause various restrictions in daily life such as housework and/or daily activities.^25^ Other central factors such as autonomic abnormalities, sympathetic activation, brain abnormalities and cytokines in chronic diseases can also cause fatigue.^7^ In addition, the mental fatigue observed in patients may cause difficulties in communicating with their neighbors.^25^ In this study, fatigue was observed in 50% of the Behçet’s patients. Energy conservation techniques and aerobic exercises to reduce fatigue can be recommended in order to minimise the influence of daily living activities and increase social participation.

In the majority of Behçet’s case studies, some degree of weakening of neurocognitive functions was described.^9^ Cognitive dysfunction was found in 77.8% of Behçet’s patients.^26^ In this study, we did not observe any cognitive impairment in Behçet’s patients. Cognitive problems are mostly observed in Neuro-Behçet’s patients.^27^ The small number of Neuro-Behçet’s patients in our study may not have been enough to show the effect.

A strong correlation was found between central sensitisation and health profile, including neuropathic pain and sleep quality, in Behçet’s patients.^28^ Increased levels of proinflammatory cytokines activate the hypothalamus, leading to neuroendocrine activity and this can disrupt sleep, eating behaviour, and mood with low energy feelings.^29^ Tascilar et al. demonstrated poor sleep quality in Behçet’s patients.^30^ Especially genital ulcer and arthritis symptoms have been shown to make it difficult to fall asleep.^30,31^ Studies have shown a worse objective sleep quality in Behçet’s patients compared to the general population.^6,32^ Yeh et al. emphasised that there is a bidirectional relationship between sleep disorders and autoimmunity and that sleep disorders are seen at a high rate in Behçet’s patients.^34^ Poor sleep quality was found in 92% of our Behçet’s patients. In a review, Chen et al. found that pilates exercises positively affect sleep quality.^35^ Sleep quality should be evaluated in Behçet’s patients and additional treatments such as relaxation exercises, clinical pilates, or yoga may be recommended to improve sleep quality.

Alexithymia is a personality disorder with cognitive dys-function and emotional impairment, and is defined as a state of alienation where the person cannot express herself/himself. Alexithymia patients have difficulty distinguishing emotions regulated in the autonomic system that causes neuroendocrine and some physical diseases.^8^ There is a strong association between inflammatory arthritis and central nervous system disorders.^8,36^ Many factors such as neurobiological disorders, central nervous system disorders such as variations in the brain neuron system or genetic effects may play a role in the aetiology of alexithymia.^8^ There is an increase in alexithymia levels in Behçet’s patients and this level is associated with depression and anxiety.^8^ Alexithymia can have adverse effects that cause extreme alertness, increase bodily sensations, and cause high levels of anxiety and depression.^37^ This may cause patients to feel their already chronic pain more. Another result of this study was that 52% of Behçet’s patients had alexithymia and 26% had possible alexithymia. According to these results, the patients may feel more pain and higher levels of anxiety and anxiety as a result of increased bodily sensations. 52% of Behçet’s patients in this study had mucocutaneous involvement. Eye and skin findings, which occur more frequently in mucocutaneous involvement, may have affected the quality of life. Studies in the related literature have shown that the presence of arthritis, eye and vascular involvement in Behçet’s patients significantly affects the quality of life.^11,23^ Oral ulcers affect the patient’s ability to eat. Ocular symptoms may cause the patient to have vision problems, trouble writing, trouble walking, and movement in intense sunlight or bright light. Fatigue makes it difficult to form social relationships and work.^24,25^ Sandikci et al. found a positive relationship between headache and quality of life in Behçet’s disease.^23^ The fact that Behçet’s is a chronic immunoinflammatory disease and arthritis in the joints causes chronic pain can negatively affect the quality of life.^11^ As a result of this study, we observed that the quality of life of 50% of Behçet’s patients was below the median value. The reason for this may be that the patients’ activities of daily living are more affected due to the high rates of mucocutaneous involvement.

In this study, the pharmacological treatments received by Behçet’s patients were divided into 3 groups (first line oral therapy, biological therapy and no therapy), and there was a significant difference between major minor organ involvement. The patients with major organ involvement were receiving more biological therapy. The reason why there was no difference between major and minor organ involvement among the evaluated parameters may be due to the higher rate of biological therapy.

The limitation of this study is that we could not compare the effects according to the disease activity level, since we did not measure the disease activity. In future studies, the relationship between disease activity and the level of exposure can be evaluated or comparisons can be made. Since the cut-off values of BETY-BQ, MAF Scale, and SF-36 were not available, this study was interpreted using median values. This may shed light on future studies to establish cut-off values for these outcome measures. Also, results regarding the comparison of Behçet’s patients according to disease type (mucocutaneous, vascular, ocular and neuro-Behçet’s) may be interesting in terms of literature. In addition, disease status such as “how many had a relapse” can be evaluated in more detail.

## CONCLUSION

In conclusion, negative effects were observed in parameters such as biopsychosocial status, fatigue, sleep quality, alexithymia, and quality of life in Behçet’s patients. The presence of major or minor organ involvement in the patients did not change these negative effects. For this reason, we recommend biopsychosocial therapies, such as the Cognitive Exercise Therapy Approach, in addition to exercise and pharmacological treatments, were included in the management of Behçet’s disease. Also, regular physical activity and exercises can be used for the management of chronic pain and psychosocial effects in patients.
